# Supplementary material for: Engineered cytosine base editor enabling broad-scope and high-fidelity gene editing in Streptomyces
Source: Nat Commun. 2024 Jul 7;15:5687. doi: 10.1038/s41467-024-49987-3 (PMC11227558; doi:10.1038/s41467-024-49987-3)
Supplement: Supplementary file 1 — Supplementary Information [file 41467_2024_49987_MOESM1_ESM.pdf]

Supplementary Information

Engineered cytosine base editor enabling broad-scope and high-fidelity gene editing in *Streptomyces*

Jian Wang[1], Ke Wang[1], Zhe Deng[1], Zhiyu Zhong[1], Guo Sun[1], Qing Mei[1], Fuling Zhou[1], Zixin Deng[1] and Yuhui Sun[1,2,*]

[1] Department of Hematology, Zhongnan Hospital of Wuhan University, School of Pharmaceutical Sciences, Wuhan University, Wuhan 430071, China

[2] School of Pharmacy, Huazhong University of Science and Technology, Wuhan 430030, China

* E-mail: yhsun@whu.edu.cn

Table of Contents

Supplementary Figures

Supplementary Fig. 1 Schematics of the sgRNA cloning strategy

Supplementary Fig. 2 Comparative analysis of editing efficiency between the BE3 system and eSCBE3-NGs based on hAPOBEC3A(Y130F)

Supplementary Fig. 3 Characterization of SCBE3-NGs and eSCBE3-NGs capable of targeting NGN PAMs

Supplementary Fig. 4 Genome- and transcriptome-wide off-target effects induced by eSCBE3-NG-HF1 or eSCBE3-NG-Hypa

Supplementary Fig. 5 Biosynthetic pathway of avermectins

Supplementary Fig. 6 Sanger sequencing results of exconjugants for *S. avermitilis* 3-115 mutants screening

Supplementary Fig. 7 LC-ESI-HRMS analysis of avermectin B1a from control strain *S. avermitilis 3-115* and oligomycin C from mutant strains ΔDH6, ΔDH8 and ΔDH9

Supplementary Fig. 8 ^{1}H NMR spectrum (600 MHz, CH_3OH-d_4) of oligomycin C

Supplementary Fig. 9 Genome-wide off-target evaluation of eSCBE3-NG-Hypa in mutants of S. avermitilis 3-115

Supplementary Fig. 10 Sanger sequencing results of exconjugants for *S. avermitilis* 3-115 mutants screening

Supplementary Fig. 11 LC-ESI-HRMS analysis of fermentation extracts from control strain *S. avermitilis* 3-115 and its mutants Δolm, Δpte, and ΔolmΔpte

Supplementary Fig. 12 Growth curves of various strains in TSB medium with propionate.

Supplementary Fig. 13 Compatibility assessment of SuperFi-Cas9 with SpCas9-NG.

Supplementary Tables

Supplementary Table 1. ^{1}H NMR data (600 MHz, CH_3OH-d_4) of oligomycin C

Supplementary Table 2. Information of secondary metabolite regions in *S. avermitilis* 3-115

Supplementary Table 3. Strains used in this study

Supplementary Note

Supplementary Note 1. Bioinformatics analysis of base editing in *S. coelicolor* M145

Supplementary References

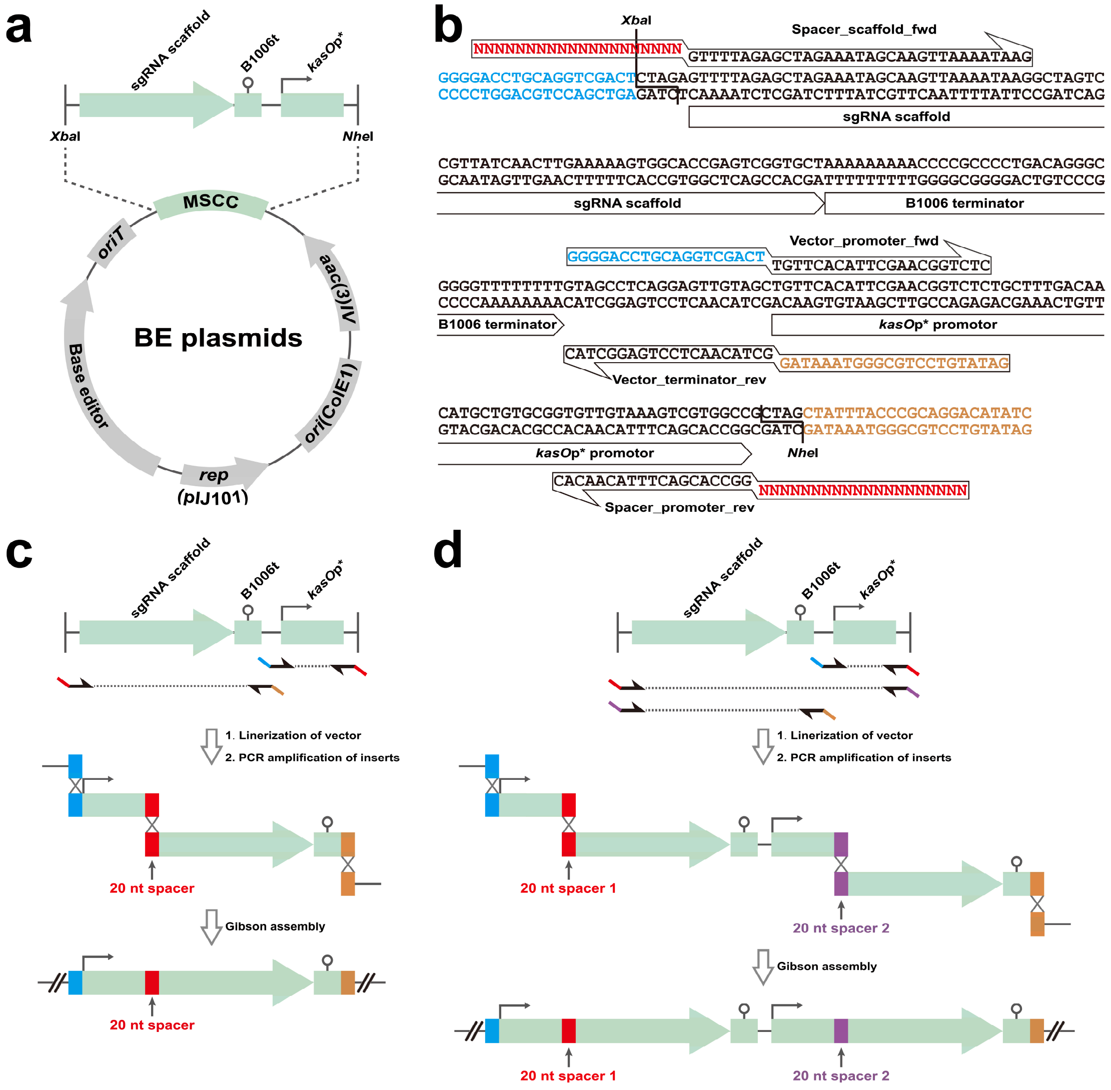

Supplementary Fig. 1 Schematics of the sgRNA cloning strategy. **a** Schematic of the *Streptomyces* base editing plasmids. MSCC, multiple sgRNA cloning cassette; *kas*Op*, *Streptomyces* constitutive promotor; B1006t, B1006 terminator; *aac(3)IV*, apramycin resistance gene; *ori*(ColE1), *E. coli* replicon; *rep*(pIJ101), *Streptomyces* replicon; *oriT*, origin of transfer derived from RK2 plasmid. **b** Complete sequence of MSCC that serves as PCR template for the amplification of Gibson assembly inserts. Red bases indicate the 20-nt spacer sequences should be introduced by primers. Blue and orange bases highlight overlap region between linear vector and PCR amplified fragments. **c**, **d** The schematic diagram shows the cloning of single (**c**) and multiple (**d**) sgRNAs, respectively. Rearrangement of the elements is required to construct the functional sgRNA expression cassettes (*kas*Op*-20-nt spacer-sgRNA scaffold-B1006t) by MSCC. Red and purple rectangles indicate the 20-nt spacer sequences should be introduced by primers; blue and orange rectangles highlight overlap regions between linear vector and PCR amplified fragments.

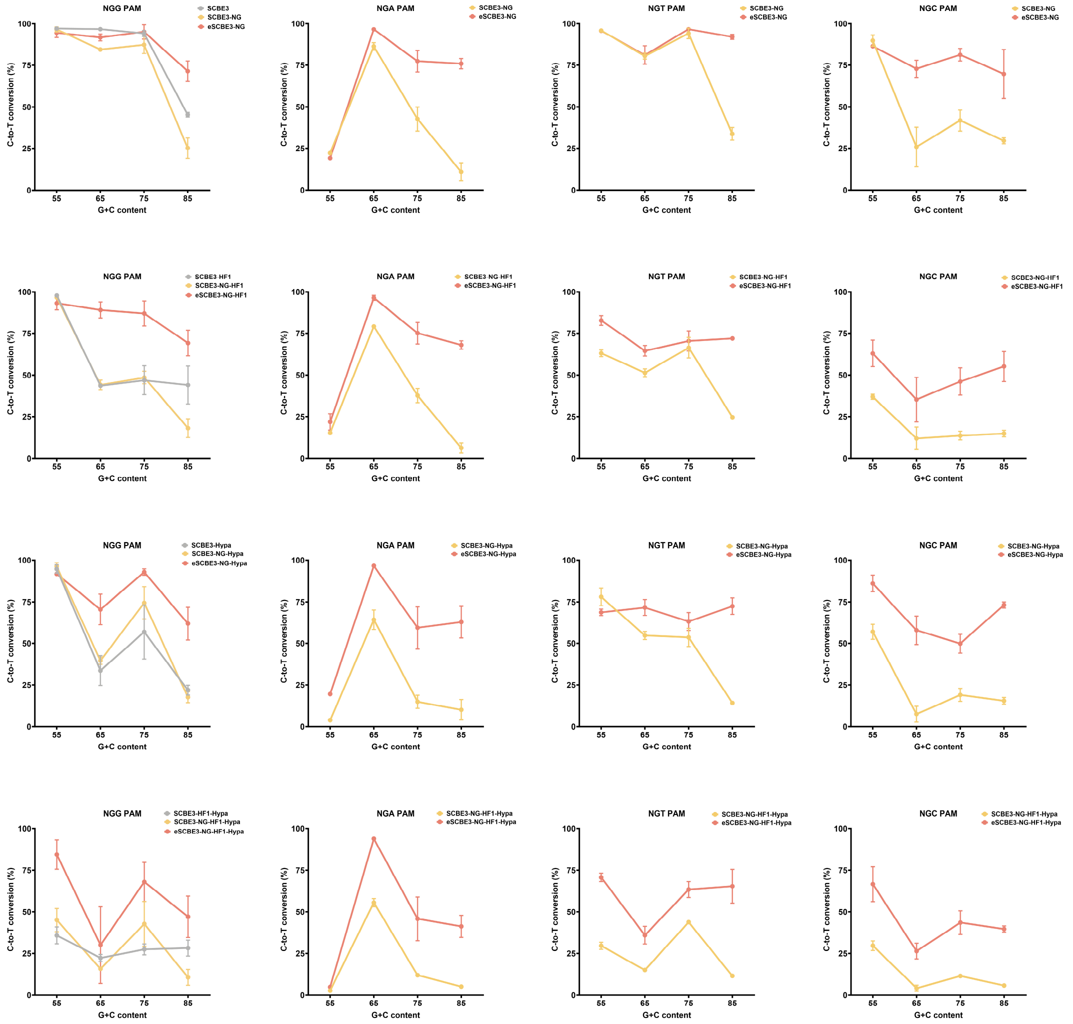

Supplementary Fig. 2 Comparative analysis of editing efficiency between the BE3 system and eSCBE3-NGs based on hAPOBEC3A(Y130F). The line graphs depict the data presented in Fig. 2*a*, with means and error bars representing the s.d. from *n* = 3 independent biological replicates.

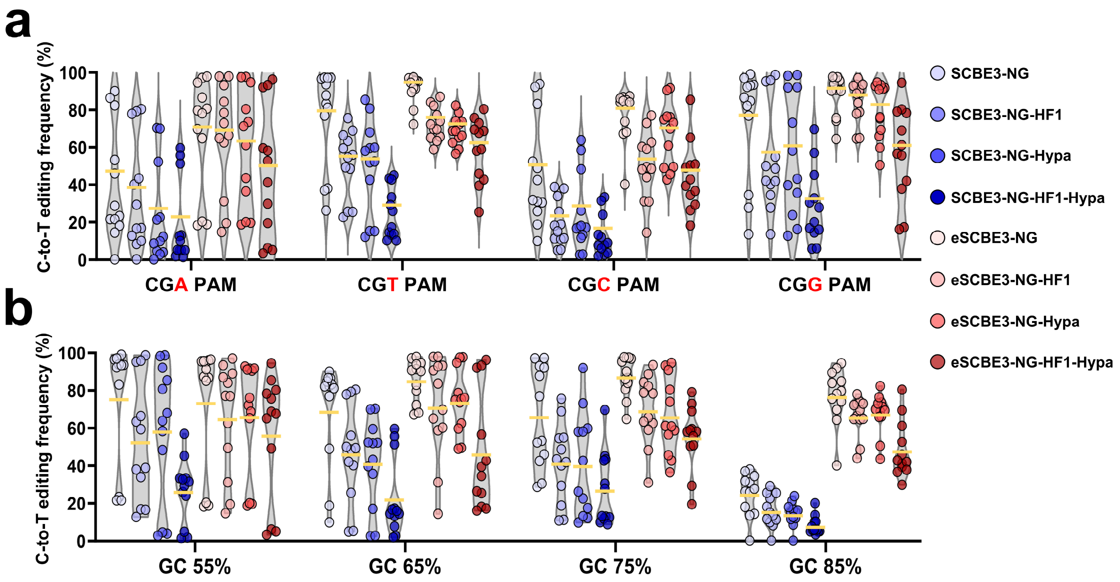

Supplementary Fig. 3 Characterization of SCBE3-NGs and eSCBE3-NGs capable of targeting NGN PAMs. **a** Summary of the results in Fig. 2a for SCBE3-NGs and eSCBE3-NGs, but grouped by the NGA, NGT, NGC and NGG PAM of protospacers. **b** Summary of the results in Fig. 2a for SCBE3-NGs and eSCBE3-NGs, but grouped by the 55%, 65%, 75%, and 85% GC content of protospacers. Mean modification (*n* = 12) of each base editor is shown as a horizontal yellow line, and the grey outline is a violin plot.

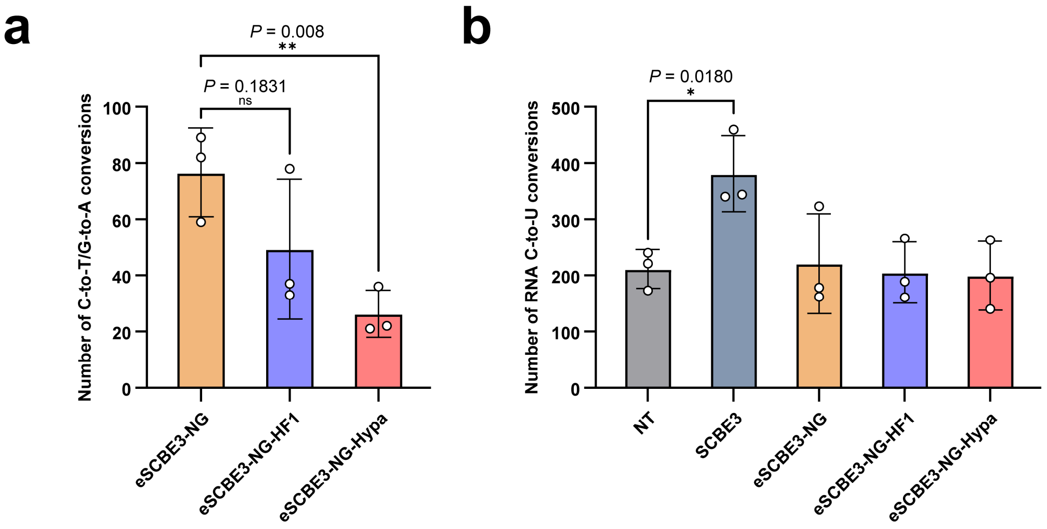

Supplementary Fig. 4 Genome- and transcriptome-wide off-target effects induced by eSCBE3-NG-HF1 or eSCBE3-NG-Hypa. **a** Number of total DNA off-target C-to-T / G-to-A conversions in Fig. 4c induced by the indicated BEs. **b** Number of total RNA off-target C-to-U conversions in Fig. 4e induced by the indicated BEs. Data are mean ± s.e.m. from three independent experiments. ns (not significant), **P* < 0.05, ***P* < 0.01. The statistical test was two-sided and no adjustments were made for multiple comparisons.

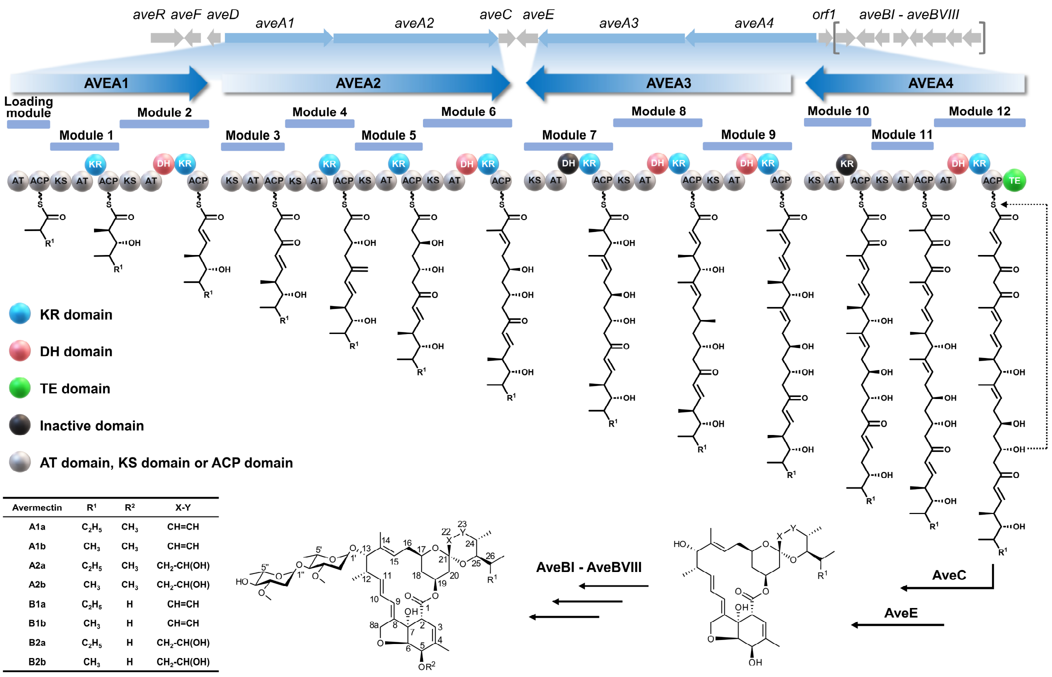

Avermectin	R^1	R^2	X-Y
A1a	C_2H_5	CH_3	CH=CH
A1b	CH_3	CH_3	CH=CH
A2a	C_2H_5	CH_3	CH_2-CH(OH)
A2b	CH_3	CH_3	CH_2-CH(OH)
B1a	C_2H_5	H	CH=CH
B1b	CH_3	H	CH=CH
B2a	C_2H_5	H	CH_2-CH(OH)
B2b	CH_3	H	CH_2-CH(OH)

Supplementary Fig. 5 Biosynthetic pathway of avermectins. Arrows indicate genetic organization of the gene cluster for avermectin biosynthesis, of which blue arrows denote avermectin PKS gene. Each circle represents an enzymatic domain in the PKS multifunctional polypeptide. AT, acyltransferase domain; DH, dehydratase domain; KR, β-ketoacyl-ACP reductase domain; KS, β-ketoacyl-ACP synthase domain; ACP, acyl carrier protein domain; TE, thioesterase domain. The black shaded domains in module 7 and module 10 have no function in polyketide-chain elongation.

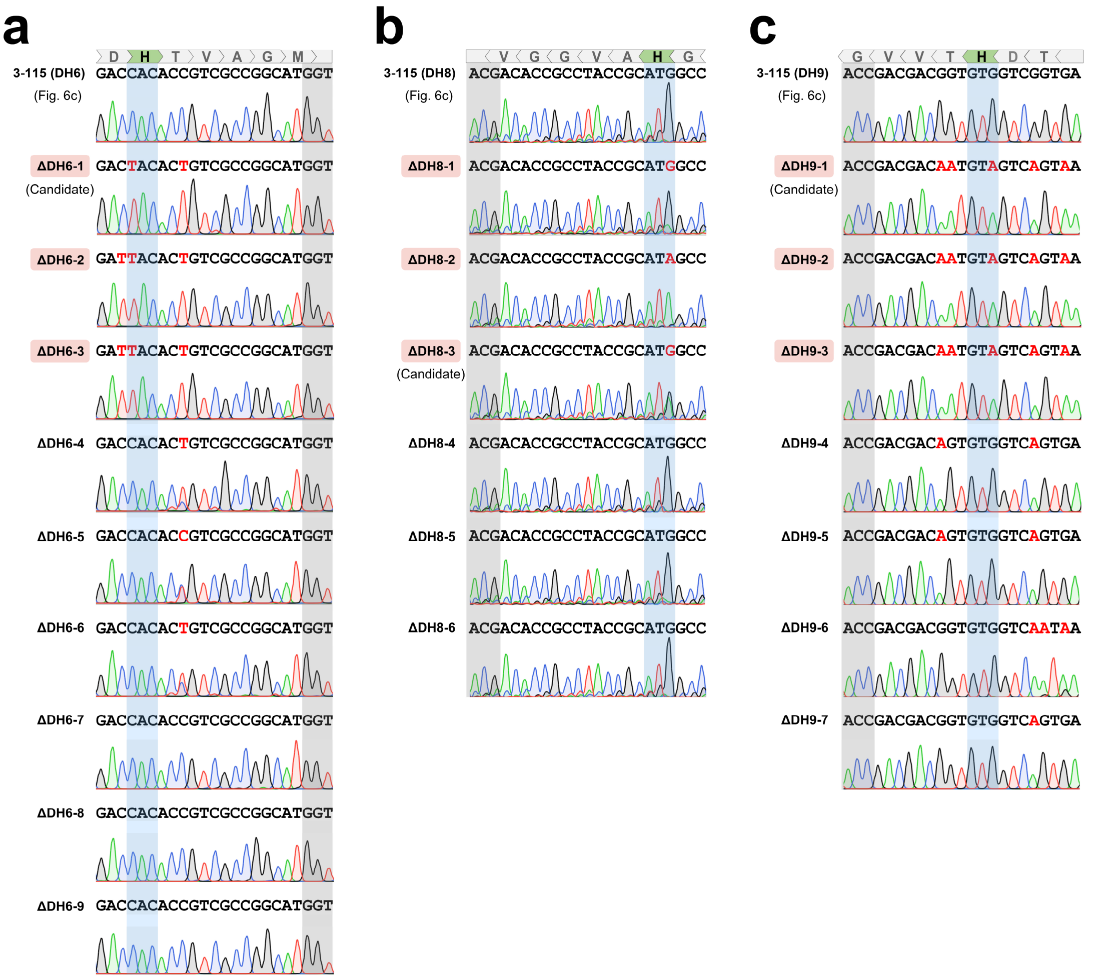

Supplementary Fig. 6 Sanger sequencing results of exconjugants for *S. avermitilis* 3-115 derivated mutants screening. **a-c** Chromatograms represent the editing events in the screening process for mutants ΔDH6, ΔDH8, and ΔDH9, respectively. The PAM sequence of the three protospacers is shaded in grey, and the codons of the active His in DH6, DH8, and DH9 are highlighted in blue shading. Red bases within the protospacers indicate converted bases or overlapping peaks in the chromatograms. Exconjugants containing the target C-to-T or G-to-A conversion are enclosed in a red rectangle, and candidate exconjugants will be subsequently undergone plasmid curing to obtain a pure mutant.

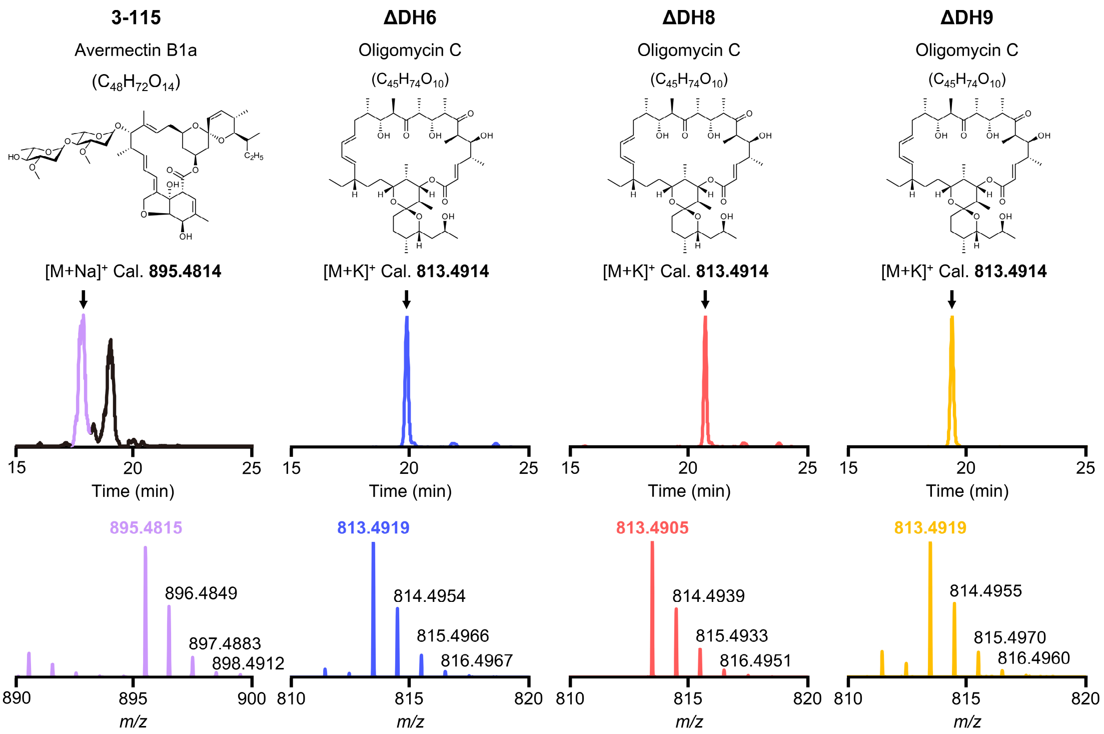

Supplementary Fig. 7 LC-ESI-HRMS analysis of avermectin B1a ($[M+Na]^+$ Cal. 895.4814) in control strain *S. avermitilis* 3-115 and oligomycin C ($[M+K]^+$ Cal. 813.4914) in mutant strains ΔDH6, ΔDH8, and ΔDH9, respectively, which related to Fig. 6d. Arrows indicate target peaks. Experiments were repeated three times independently with similar results.

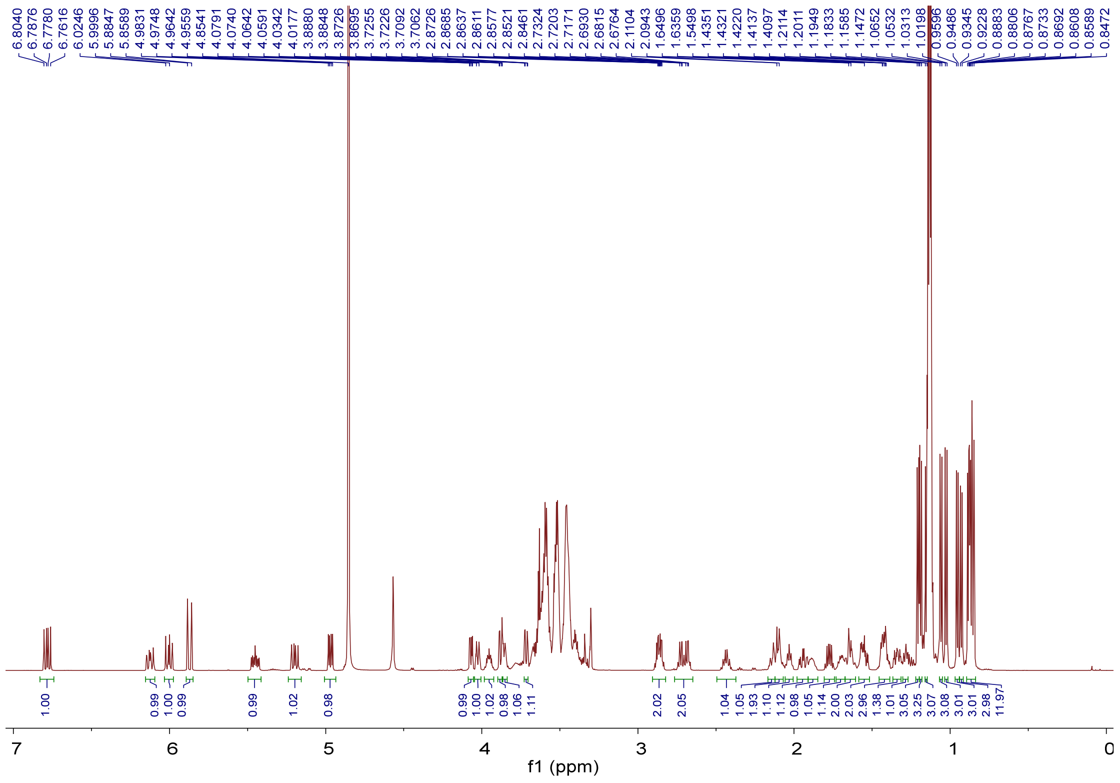

Supplementary Fig. 8 ^{1}H NMR spectrum (600 MHz, CH_3OH-d_4) of oligomycin C.

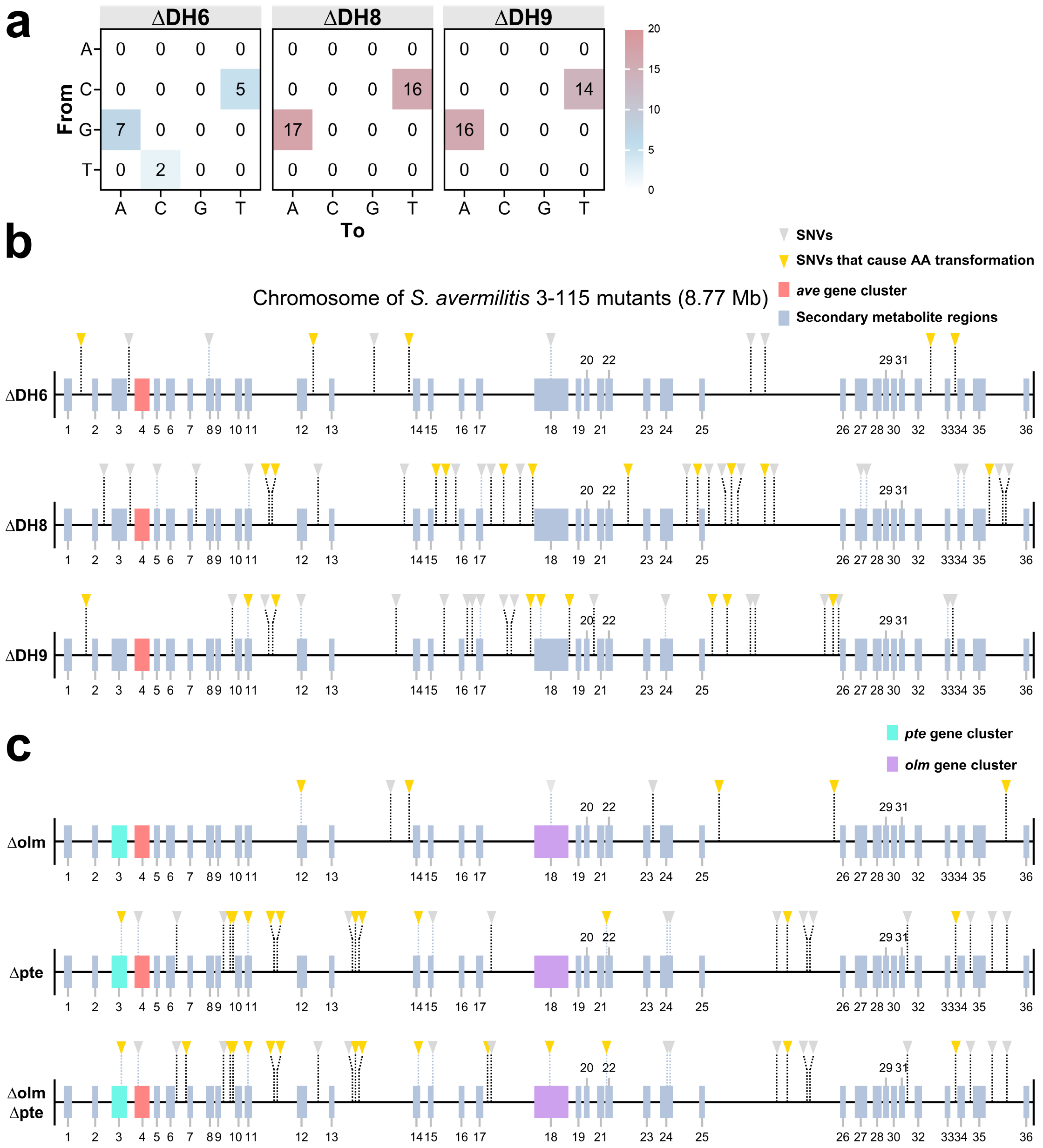

Supplementary Fig. 9 Genome-wide off-target evaluation of eSCBE3-NG-Hypa in mutants of *S. avermitilis* 3-115. **a** The heat map displays the distribution of nucleotide changes in mutants ΔDH6, ΔDH8, and ΔDH9 compared to the reference genome of *S. avermitilis* 3-115. **b** Illustration depicting the distribution of SNVs in the genome of *S. avermitilis* 3-115 for its mutants ΔDH6, ΔDH8, and ΔDH9. **c** Illustration presenting the distribution of SNVs in the genome of *S. avermitilis* 3-115 for its mutants Δolm, Δpte, and ΔolmΔpte. Rectangles in (**b**) and (**c**) indicate regions associated with secondary metabolites, and additional details can be found in Supplementary Table 2.

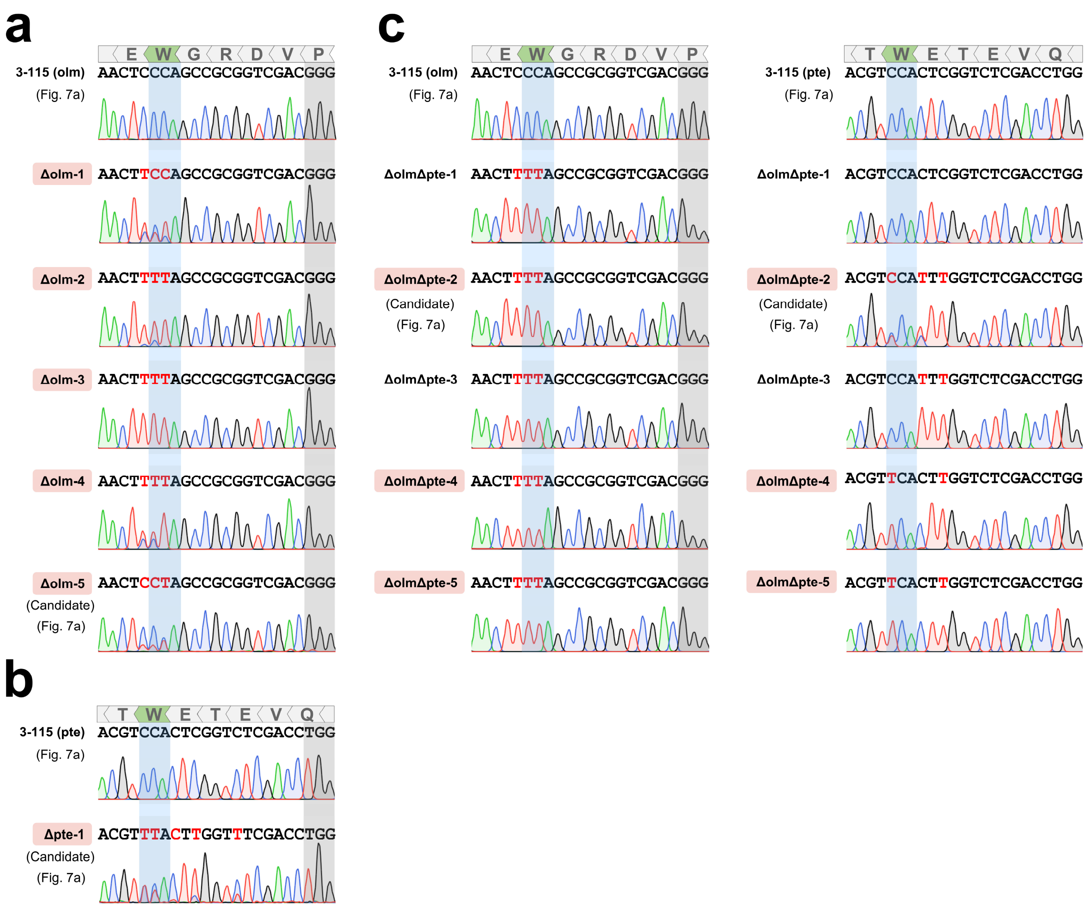

Supplementary Fig. 10 Sanger sequencing results of exconjugants for *S. avermitilis* 3-115 derivated mutants screening. **a-c** Chromatograms represent the editing events in the screening process for mutants Δolm, Δpte, and ΔolmΔpte, respectively. The PAM sequence of the three protospacers is shaded in grey, and the codons of the target tryptophan (W) are highlighted in blue shading. Red bases within the protospacers indicate converted bases or overlapping peaks in the chromatograms. Exconjugants containing the target C-to-T / G-to-A conversion are enclosed in a red rectangle, and candidate exconjugants will be subsequently undergone plasmid curing to obtain a pure mutant.

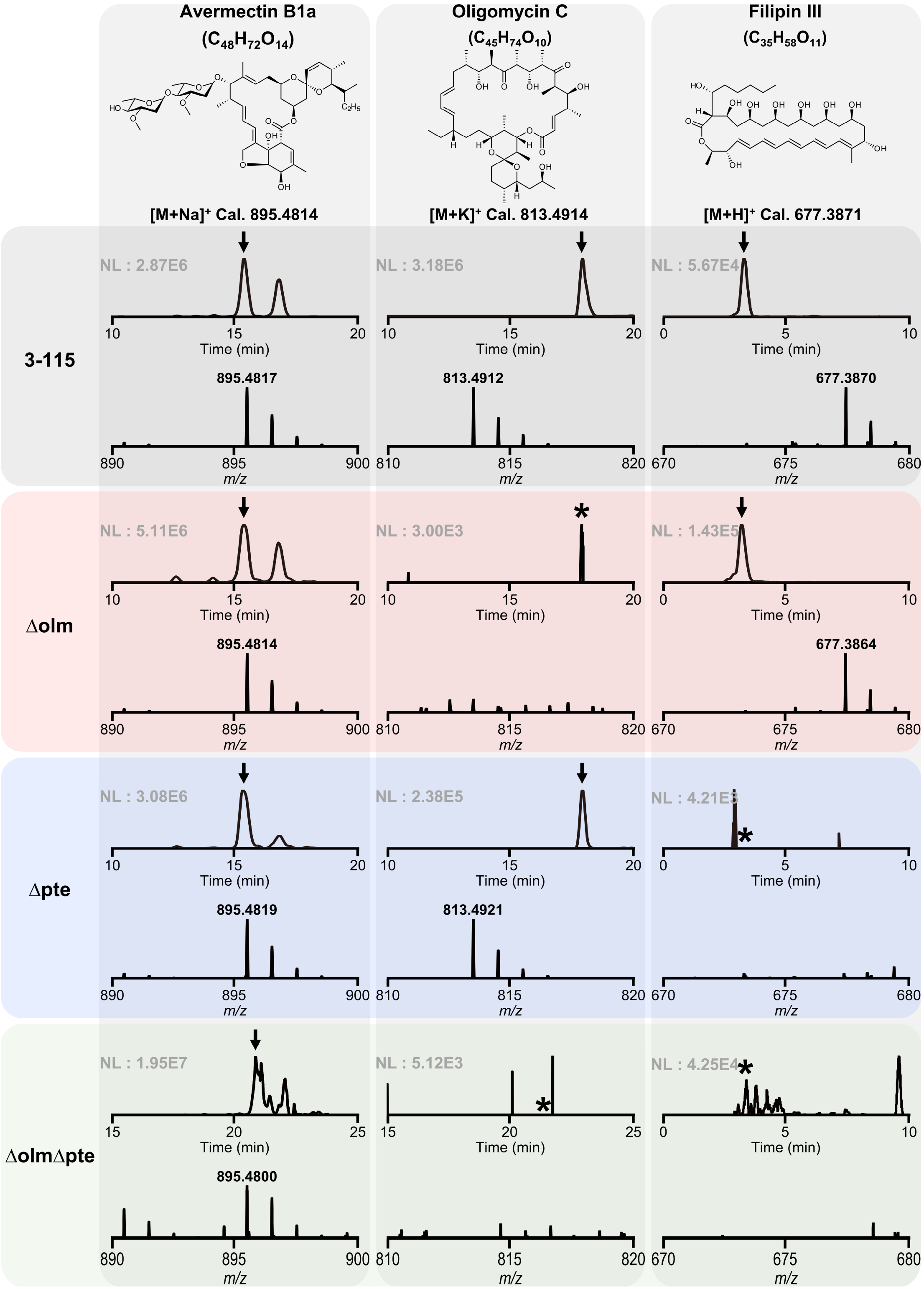

Supplementary Fig. 11 LC-ESI-HRMS analysis of fermentation extracts from control strain *S. avermitilis* 3-115 and its mutants Δolm, Δpte, and ΔolmΔpte. Corresponding compounds detected or not are indicated by the arrows or asterisks. Experiments were repeated three times independently with similar results.

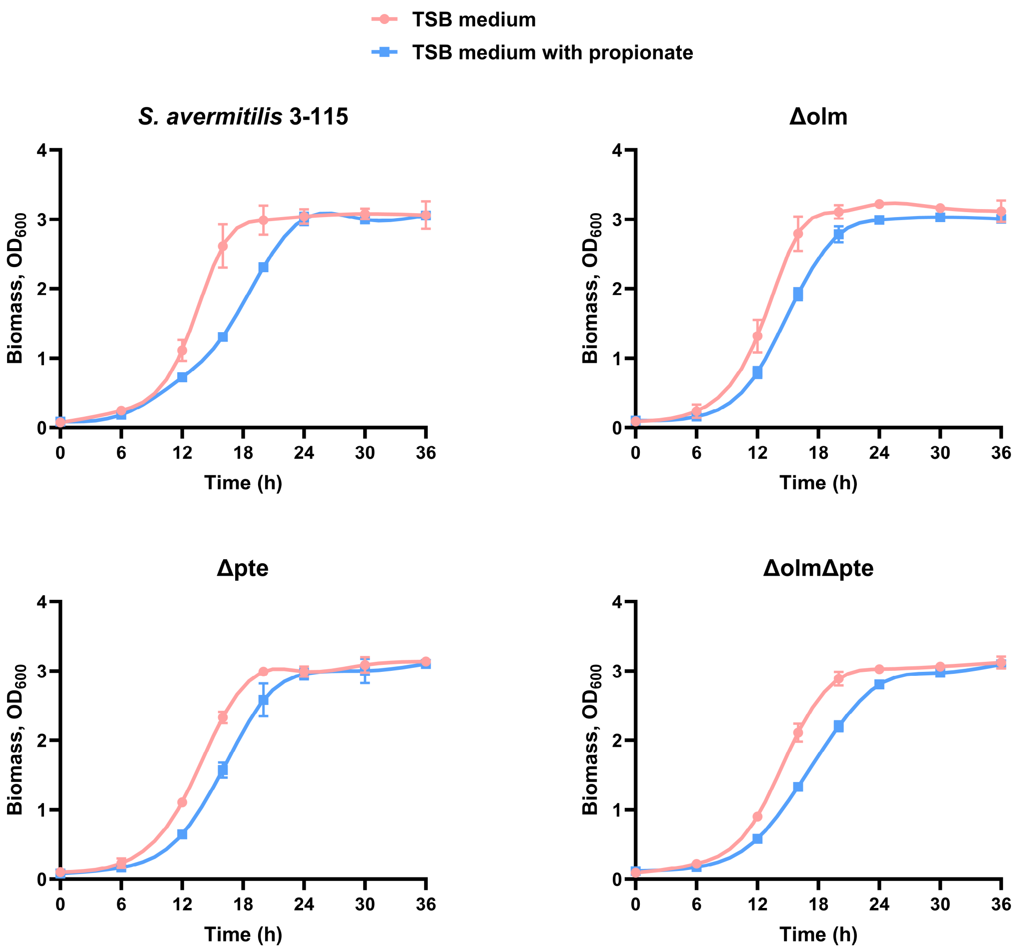

Supplementary Fig. 12 Growth curves of various strains in TSB medium with propionate. Propionate derivatives, including propionyl-CoA and methylmalonyl-CoA, induce growth inhibition in *S. avermitilis* 3-115 and its corresponding mutants Δolm, Δpte, and ΔolmΔpte. Growth curves of various strains in TSB medium are presented by red connecting lines, while those cultured in TSB medium with 1 g/L propionate are indicated by blue connecting lines. Mean and s.d. shown for n = 3 biologically independent samples.

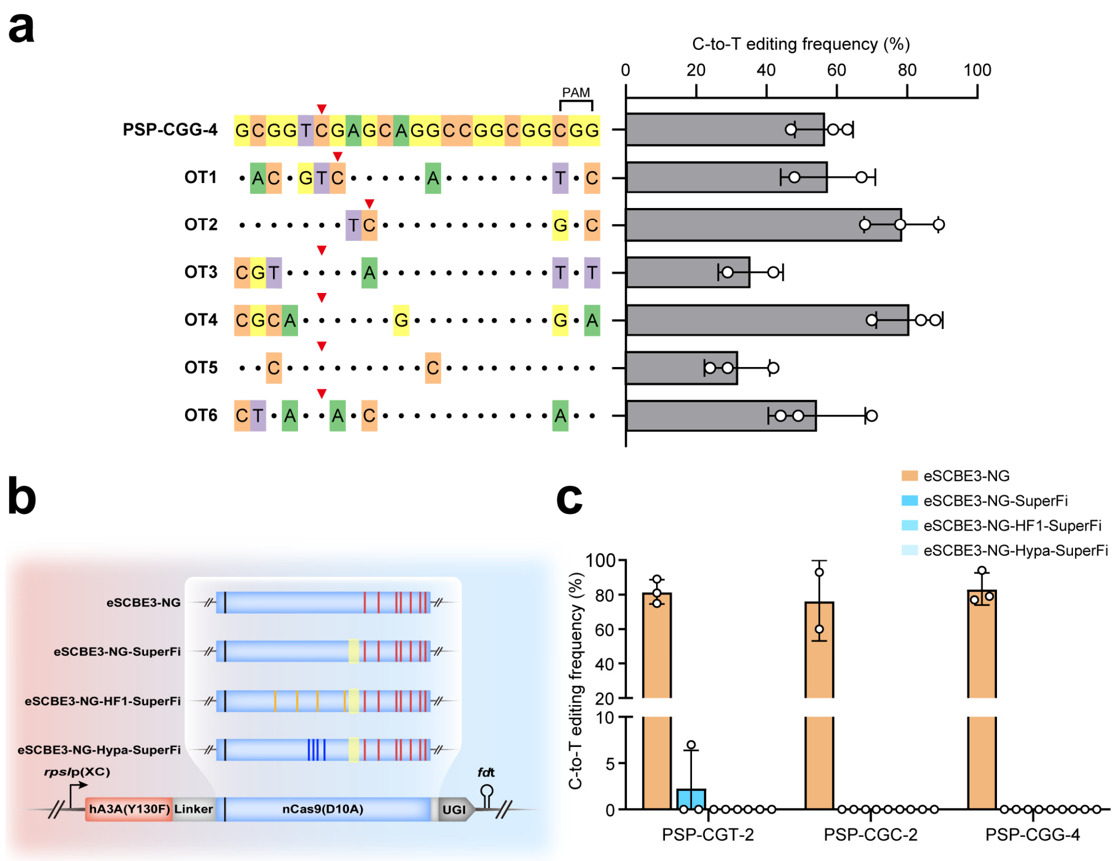

Supplementary Fig. 13 Compatibility assessment of SuperFi-Cas9 with SpCas9-NG. **a** Sequence details of off-target sites exhibiting high C-to-T editing frequencies induced by eSCBE3-NG-Hypa. Data are extracted from Fig. 4b and Supplementary Table 2. Red triangles highlight edited C. **b** Schematic representation of the construction of SuperFi-Cas9-based eSCBE3-NGs. In the portion of nCas9(D10A), vertical red lines signify mutation sites R1335V / L1111R / D1135V / G1218R / E1219F / A1322R / T1337R of SpCas9-NG; vertical yellow lines denote mutation sites N497A / R661A / Q695A / Q926A of SpCas9-HF1; vertical blue lines represent mutation sites N692A / M694A / Q695A / H698A of HypaCas9, and the yellow region indicates mutation sites Y1010D / Y1013D / Y1016D / V1018D / R1019D / Q1027D / K1031D of SuperFi-Cas9. **c** Editing efficiency of SuperFi-Cas9-based eSCBE3-NGs. The percentage of base conversion was estimated by EditR, a software to quantify the base editing efficiency from Sanger sequencing chromatograms[1]. All bar graph shows means and error bars representing s.d. of n = 2 or n = 3 independent biological replicates.

Supplementary Table 1. ^{1}H NMR data (600 MHz, CH_3OH-*d*$_4$) of oligomycin C

No.	δ_H
1	—
2	5.87 (d, 15.5 Hz, 1H)
3	6.78 (dd, 15.6, 9.8 Hz, 1H)
4	2.43 (tq, 9.4, 6.7 Hz, 1H)
5	3.88 (dd, 9.2, 1.9 Hz, 1H)
6	2.72 (qd, 7.3, 1.9 Hz, 1H)
7	—
8	2.68 (qd, 7.0, 3.2 Hz, 1H)
9	4.07 (dd, 8.9, 3.0 Hz, 1H)
10	2.86 (dq, 9.8, 6.9 Hz, 1H)
11	—
12	2.86 (dq, 9.8, 6.9 Hz, 1H)
13	3.72 (dd, 9.8, 1.8 Hz, 1H)
14	1.70 (m, 1H)
15a/b	2.11 (m, 1H), 2.09 (m, 1H)
16	5.45 (ddd, 14.9, 10.1, 4.7 Hz, 1H)
17	6.13 (dd, 15.1, 10.5 Hz, 1H)
18	6.13 (dd, 15.1, 10.5 Hz, 1H)
19	5.20 (dd, 15.0, 9.6 Hz, 1H)
20	1.88 (m, 1H)
21a/b	1.64 (m, 1H), 1.43 (m,1H)
22a/b	1.64 (m, 1H), 1.05 (m,1H)
23	3.86 (dt, 9.7, 2.6 Hz, 1H)
24	2.04 (m, 1H)
25	4.97 (dd, 11.3, 5.0 Hz, 1H)
26	1.77 (dd, 11.3, 6.6 Hz, 1H)
27	—
28a/b	1.94 (td, 13.6, 4.5 Hz, 1H), 1.16 (m, 1H)
29a/b	2.15 (m, 1H), 1.43 (m,1H)
30	1.57 (m, 1H)
31	4.03 (dt, 10.0, 2.7 Hz, 1H)
32a/b	1.55 (m, 1H), 1.34 (ddd, 13.9, 9.1, 2.9 Hz, 1H)
33	3.95 (dqd, 9.7, 6.2, 3.8 Hz, 1H)
34	1.21 (d, 6.2 Hz, 3H)
35	1.19 (d, 6.9 Hz, 3H)
36	1.06 (d, 7.2 Hz, 3H)
37	0.88 (d, 6.9 Hz, 3H)
38	1.15 (d, 6.8 Hz, 3H)
39	0.87 (d, 6.8 Hz, 3H)
40	1.03 (d, 6.9 Hz, 3H)
41a/b	1.43 (m,1H), 1.29 (m, 1H)
42	0.86 (t, 7.4 Hz, 3H)
43	0.85 (d, 7.0 Hz, 3H)
44	0.95 (d, 6.6 Hz, 3H)
45	0.93 (d, 7.0 Hz, 3H)

Supplementary Table 2. Information of secondary metabolite regions in *S. avermitilis* 3-115

Region	Type	From	To	Putative metabolite	Similarity with known cluster
Region 1	Terpene, T1 PKS	70,787	133,384	Avermitilol	100%
Region 2	Lassopeptide	331,161	353,549	Cattlecin	75%
Region 3	T1 PKS	519539	627,710	Filipins	100%
Region 4	T1 PKS	722,832	827,528	Avermectins	100%
Region 5	Terpene	882,423	907,733	Carotenoid	100%
Region 6	NRPS-like, T1 PKS	980,545	1,028,926	Lasalocid	3%
Region 7	Lanthipeptide-class-iii, Ripp-like	1,204,241	1,231,689	Informatipeptin	100%
Region 8	hglE-KS, T1 PKS	1,348,378	1,400,049	Hexacosalactone A	11%
Region 9	NI-siderophore	1,408,329	1,440,001	Peucechelin	25%
Region 10	T1 PKS	1,579,997	1,624,550	-	-
Region 11	T3 PKS	1,645,668	1,686,726	Flaviolin /1,3,6,8-Tetrahydroxynaphthalene	100%
Region 12	NAPAA	2,205,948	2,239,823	ε-Poly-L-lysine	100%
Region 13	Ectoine	2,481,610	2,492,014	Ectoine	100%
Region 14	Lassopeptide	3,266,170	3,288,741	Citrulassin D	100%
Region 15	Aminopolycarboxylic-acid	3,357,512	3,370,972	EDHA	88%
Region 16	Melanin	3,653,047	3,663,613	Melanin	80%
Region 17	NI-siderophore	3,759,586	3,789,358	Desferrioxamin B / Desferrioxamine E	100%
Region 18	T2 PKS, T1 PKS	4,404,955	4,602,236	Oligomycins	100%
Region 19	Terpene	4,700,551	4,721,561	Pentalenolactone	100%
Region 20	Terpene	4,736,001	4,756,924	Albaflavenone	100%
Region 21	NRPS-like, NRPS	4,866,160	4,913,478	Gobichelin A / Gobichelin B	16%
Region 22	NRPS, NRPS-like	4,916,067	4,968,164	WS9326	7%
Region 23	PKS-like, Butyrolactone	5,291,316	5,332,401	5-isoprenylindole-3-Carboxylate β-D-glycosyl ester	42%
Region 24	NRPS, Arylpolyene	5,435,906	5,514,694	Kitacinnamycin A / Kitacinnamycin B / Kitacinnamycin C / Kitacinnamycin D / Kitacinnamycin E / Kitacinnamycin F	30%
Region 25	Terpene	5,787,629	5,808,642	-	-
Region 26	NI-siderophore	7,133,936	7,163,996	Kinamycin	13%
Region 27	T2 PKS, PKS-like, T1 PKS	7,222,535	7,295,043	Auroramycin	20%
Region 28	Other, T1 PKS, PKS-like	7,341,836	7,399,659	Tambjamine BE-18591	17%
Region 29	RiPP-like	7,449,738	7,461,183	-	-
Region 30	Lassopeptide	7,470,276	7,492,721	Ashimide A / Ashimide B	10%
Region 31	Terpene	7,505,775	7,527,952	Geosmin	100%
Region 32	NI-siderophore	7,694,543	7,725,654	Paulomycin	13%
Region 33	Hydrogen-cyanide	8,031,648	8,044,635	Aborycin	28%
Region 34	Terpene	8,116,640	8,143,295	Hopene	92%
Region 35	T1 PKS	8,223,498	8,277,987	Foxicin A / Foxicin B / Foxicin C	12%
Region 36	Melanin	8,723,578	8,733,934	Melanin	100%

Supplementary Table 3. Strains used in this study

Strains / Plasmids / Primers	Features	Sources
E. coli		
DH10B	*lacZ mcrA mcrBC mrr hsd* RMS	(2)
ET12567/pUZ8002	*rec*^{F} *dam dcm Cml*^{R} *Str*^{R} *Tet*^{R} *Km*^{R}	(3)
Streptomyces		
S. coelicolor M145	*Streptomyces coelicolor* A3(2) SCP1-, SCP2-	(4)
S. avermitilis 3-115	An industrial strain with high-yield of avermectins	(5)
ΔDH6	*S. avermitilis* 3-115 derivated mutant with inactive Tyr converted from active His within the DH6 domain of the avermectin PKS	This study
ΔDH8	*S. avermitilis* 3-115 derivated mutant with inactive Tyr converted from active His within the derivated DH8 domain of the avermectin PKS	This study
ΔDH9	*S. avermitilis* 3-115 derivated mutant with inactive Tyr converted from active His within the DH9 domain of the avermectin PKS	This study
Δolm	*S. avermitilis* 3-115 derivated mutant with inactive *olm* gene cluster resulting from the introduction of premature stop codon in *olmA1*	This study
Δpte	*S. avermitilis* 3-115 derivated mutant with inactive *pte* gene cluster resulting from the introduction of premature stop codon in *pteA1*	This study
ΔolmΔpte	*S. avermitilis* 3-115 derivated mutant with dual inactive *olm* and *pte* gene clusters resulting from the introduction of premature stop codon in *olmA1* and *pteA1*, respectively	This study

Supplementary Note 1. A modified python script based on the prior study[6] for identify the protospacers enabling the introduction of premature codons in *S. coelicolor* M145 genome was shown below[7].

```
from Bio import SeqIO`
from Bio.SeqRecord import SeqRecord
import pandas as pd

def process_genome_data(gff_file, fasta_file):
    # Open GFF file and initialize variables
    x=open(gff_file)
    y = 0
    features = []
    for i in x:
        if not i.startswith('#'):
            G_objects = i.split('\t')
            if G_objects[2] == 'CDS':
                y += 1
                featuresL = G_objects[8].split(';')
                for i in featuresL:
                    featuresD = {items.split('=')[0]: items.split('=')[1] for items in featuresL}
                    featuresD['start'] = G_objects[3]
                    featuresD['end'] = G_objects[4]
                    featuresD['derection'] = G_objects[6]
                    featuresD['chrome'] = G_objects[0]
                features.append(featuresD)
    # Extract sequences from FASTA file
    fasta_dict = {}
    for seq_record in SeqIO.parse(fasta_file, "fasta"):
        fasta_dict[seq_record.id] = seq_record.seq
    return features, fasta_dict

def process_cds_data(features, fasta_dict):
    cds_dict = {}
    cds_num = 0
    for CDSd in features:
        cds_num += 1
        gene_name = CDSd['locus_tag']
        cds = SeqRecord(fasta_dict[CDSd['chrome']][int(CDSd['start']) - 21:int(CDSd['end']) +
21])
        process_sequences(CDSd, cds, gene_name, cds_dict)
    return cds_dict

def process_sequences(CDSd, cds, gene_name, cds_dict):
```

```
# Initialize variables
n20_list = []
pam_list = []
edit_codon = []
coding_strand = []
dist_to_pam = []
protein_position = []
GC_percentage=[]
GCmotif =0
GC_75_count=0
read_box=0
onlyGC_count=0
# Process "+" and "-" sequences
if CDSd['derection'] == '+':
    coding = str(cds.seq)
    non_coding = str(cds.reverse_complement().seq)
elif CDSd['derection'] == '-':
    non_coding = str(cds.seq)
    coding = str(cds.reverse_complement().seq)
else:
    print("derection error")
for aa_pos, i in enumerate(range(21, len(coding) - 21, 3)):
    codon = coding[i : i + 3]
    if codon in ["CAA", "CAG", "CGA"]:
        read_box +=1
        # allow a 4bp editing window
        for idx, p in enumerate(
            [
                coding[i - 3 : i + 20], # 17
                coding[i - 4 : i + 19], # 16
                coding[i - 5 : i + 18], # 15
                coding[i - 6 : i + 17], # 14
                coding[i - 7 : i + 16], # 13
            ]
        ):
            PAM = p[-3:]
            # NGG PAM List
            NGG_PAM_P =["AGG","TGG","CGG","GGG"]
            # NGN PAM List
            NGN_PAM =["AGA","AGT","AGC","AGG",
                      "TGA","TGT","TGC","TGG",
                      "CGA","CGT","CGC","CGG",
                      "GGA","GGT","GGC","GGG"]
            if PAM in NGG_PAM_P:
```

```
                # distance betwwen Edit-window and PAM (13-17)
                    if 17-idx in [17,16,15,14,13]:
                        n20_list.append(p[:-3])
                        gc_percentage = (p[:-3].count('G') + p[:-3].count('C')) / 20 * 1.00
                        GC_percentage.append(gc_percentage)
                        if gc_percentage >0.75:
                            if 'GC' in p[:-3][2:8]:
                                GC_75_count +=1
                        if 'GC' in p[:-3][2:8]:
                            onlyGC_count+=1
                        pam_list.append(p[-3:])
                        edit_codon.append(codon)
                        coding_strand.append("+")
                        dist_to_pam.append(17 - idx)
                        protein_position.append(aa_pos + 1)
for aa_pos, i in enumerate(range(21, len(non_coding) - 21, 3)):
    codon = non_coding[i : i + 3]
    if codon == "CCA":
        read_box +=1
        for idx, p in enumerate(
            [
                #non_coding[i : i + 23], #19
                non_coding[i - 3 : i + 20],
                non_coding[i - 4 : i + 19],
                non_coding[i - 5 : i + 18],
                non_coding[i - 6 : i + 17],
                non_coding[i - 7 : i + 16],
            ]
        ):
            PAM = p[-3:]
            NGG_PAM_N =["GGA","GGT","GGC","GGT"]
            NGN_PAM =["AGA","AGT","AGC","AGG",
                      "TGA","TGT","TGC","TGG",
                      "CGA","CGT","CGC","CGG",
                      "GGA","GGT","GGC","GGG"]
            if PAM in NGG_PAM_N:
                if 17-idx in [17,16,15,14,13]:
                    n20_list.append(p[:-3])
                    gc_percentage = (p[:-3].count('G') + p[:-3].count('C')) / 20 * 1.00
                    GC_percentage.append(gc_percentage)
                    if gc_percentage >0.75:
                        if 'GC' in p[:-3][2:8]:
                            GC_75_count +=1
```

```
                                        #GCmotif+=sum(1 for i in range(len(p[:-3]) - 1) if p[:-3][i:i+2]
== 'GC' and 2 <= i <= 8)
                                    if 'GC' in p[:-3][2:8]:
                                        onlyGC_count+=1
                                    pam_list.append(p[-3:])
                                    edit_codon.append(codon)
                                    coding_strand.append("-")
                                    dist_to_pam.append(17 - idx)
                                    protein_position.append(((len(non_coding) - 42) // 3) - aa_pos)
    print (len(GC_percentage))
    if len(GC_percentage) !=0:
        GC75p = sum(1 for num in GC_percentage if num > 0.75) / len(GC_percentage)    *
1.00
        GC75motif=GC_75_count/len(GC_percentage)
    else:
        GC75p=0
        GC75motif=0
    N20_count = len(n20_list)
    GC75p_count =sum(1 for num in GC_percentage if num > 0.75)
    cds_dict[gene_name] = {
        "N20": n20_list,
        "PAM": pam_list,
        "Edit_codon": edit_codon,
        "strand": coding_strand,
        "dist_to_pam": dist_to_pam,
        "protein_pos": protein_position,
        "GC75p": GC75p,
        "GCpercent": GC_percentage,
        "GCmotif": GC75motif,
        "N20_count":N20_count,
        "GC75p_count":GC75p_count,
        "GCmotif_count":GC_75_count,
        "onlyGC_count":onlyGC_count
    }
    return cds_dict

def main(gff_file, fasta_file):
    # Extract CDS information from GFF file
    # Extract sequences from FASTA file\
    features, fasta_dict = process_genome_data(gff_file, fasta_file)
    # Process CDS data
    cds_dict = process_cds_data(features, fasta_dict)
  # Convert dictionary to a Pandas DataFrame and write to Excel
    df = pd.DataFrame.from_dict(cds_dict, orient='index')
```

```
    df.to_excel('NGG_CDS_codon1317_2GC.xlsx')
if __name__ == "__main__":
# Usage example:
# Choose the appropriate PAM List in the line 93-95 and 142-143.
    main('GCF_000203835.1_ASM20383v1_genomic.gff', 'Genome of S. coelicolor
M145.fasta')
```

Supplementary References

1. Kluesner, M. G. et al. EditR: a method to quantify base editing from Sanger sequencing. *CRISPR J.* **1**, 239–250 (2018).
2. Grant, S. G., Jessee, J., Bloom, F. R. & Hanahan, D. Differential plasmid rescue from transgenic mouse DNAs into *Escherichia coli* methylation-restriction mutants. *Proc. Natl. Acad. Sci. USA.* **87**, 4645–4649 (1990).
3. MacNeil D. J. et al. Analysis of *Streptomyces avermitilis* genes required for avermectin biosynthesis utilizing a novel integration vector. *Gene* **111**, 61–68 (1992).
4. Hopwood, D. A., Kieser, T., Bibb, M., Buttner, M. & Chater, K. F. *Practical Streptomyces Genetics* (John Innes Foundation, 2000).
5. Gao, H. et al. Identification of avermectin-high-producing strains by high-throughput screening methods. *Appl. Microbiol. Biotechnol.* **85**,1219–1225 (2010).
6. Xia, Y. et al. The construction of a PAM-less base editing toolbox in *Bacillus subtilis* and its application in metabolic engineering. *Chem. Eng. J.* **469**, 143865 (2023).
7. Wang, J. & Deng, Z. A python script for identify the protospacers enabling the introduction of premature codons in *S. coelicolor* M145 genome. Zenodo. https://doi.org/10.5281/zenodo.11579018 (2024).
