# Supplementary material for: Engineered cytosine base editor enabling broad-scope and high-fidelity gene editing in Streptomyces
Source: Nat Commun. 2024 Jul 7;15:5687. doi: 10.1038/s41467-024-49987-3 (PMC11227558; doi:10.1038/s41467-024-49987-3)
Supplement: Supplementary file 3 — Description of Additional Supplementary Files [file 41467_2024_49987_MOESM3_ESM.pdf]

Description of Additional Supplementary Files

File Name: Supplementary Data 1
Description: Information of protospacers, base editors, plasmids and primers used in this study.

File Name: Supplementary Data 2
Description: Information of bioinformatics analysis related to Fig. 3.

File Name: Supplementary Data 3
Description: Information of identified SNVs at genomic and transcriptomic level in *S. coelicolor* M145.

File Name: Supplementary Data 4
Description: Information of identified SNVs at genomic level in *S. avermitilis* 3-115 mutants.

File Name: Supplementary Data 5
Description: Information of amplicon sequencing data.
