# Supplementary material for: Engineered cytosine base editor enabling broad-scope and high-fidelity gene editing in Streptomyces
Source: Nat Commun. 2024 Jul 7;15:5687. doi: 10.1038/s41467-024-49987-3 (PMC11227558; doi:10.1038/s41467-024-49987-3)
Supplement: Supplementary file 9 — Reporting Summary [file 41467_2024_49987_MOESM9_ESM.pdf]

nature portfolio

Corresponding author(s): Yuhui Sun

Last updated by author(s): Jun 12, 2024

Reporting Summary

Nature Portfolio wishes to improve the reproducibility of the work that we publish. This form provides structure for consistency and transparency in reporting. For further information on Nature Portfolio policies, see our Editorial Policies and the Editorial Policy Checklist.

Statistics

For all statistical analyses, confirm that the following items are present in the figure legend, table legend, main text, or Methods section.

n/a	Confirmed	
☐	☒	The exact sample size (*n*) for each experimental group/condition, given as a discrete number and unit of measurement
☐	☒	A statement on whether measurements were taken from distinct samples or whether the same sample was measured repeatedly
☐	☒	The statistical test(s) used AND whether they are one- or two-sided<br>*Only common tests should be described solely by name; describe more complex techniques in the Methods section.*
☒	☐	A description of all covariates tested
☒	☐	A description of any assumptions or corrections, such as tests of normality and adjustment for multiple comparisons
☐	☒	A full description of the statistical parameters including central tendency (e.g. means) or other basic estimates (e.g. regression coefficient) AND variation (e.g. standard deviation) or associated estimates of uncertainty (e.g. confidence intervals)
☐	☒	For null hypothesis testing, the test statistic (e.g. *F*, *t*, *r*) with confidence intervals, effect sizes, degrees of freedom and *P* value noted<br>*Give P values as exact values whenever suitable.*
☒	☐	For Bayesian analysis, information on the choice of priors and Markov chain Monte Carlo settings
☒	☐	For hierarchical and complex designs, identification of the appropriate level for tests and full reporting of outcomes
☒	☐	Estimates of effect sizes (e.g. Cohen's *d*, Pearson's *r*), indicating how they were calculated

Our web collection on statistics for biologists contains articles on many of the points above.

Software and code

Policy information about availability of computer code

Data collection	Amplicon sequencing was carried out by AZENTA Life Sciences and sequenced on Illumina Miseq.<br>Whole genome sequencing and whole genome re-sequencing were carried out by AZENTA Life Sciences and sequenced on Illumina Hiseq X.<br>RNA sequencing was performed at Majorbio Bio-pharm Biotechnology Co., Ltd and sequenced on Illumina NovaSeq 6000.<br>Sequences displayed in this study was Sanger sequenced by Tsingke Biotech.<br>A python script for identify the protospacers enabling the introduction of premature codons in S. coelicolor M145 genome: https://doi.org/10.5281/zenodo.11579018.
Data analysis	SnapGene version 4.3.6 for analyzing Sanger sequencing results.<br>GraphPad Prism version 10.1.2 for drawing figures and calculating P values .<br>Origin 2021 version 9.95 for drawing error band graph.<br>CRISPResso2 (https://github.com/pinellolab/CRISPResso2) for calculating the base editing efficiency from NGS data.<br>EditR (https://moriaritylab.shinyapps.io/editr_v10/) for quantifying the base editing efficiency from Sanger sequencing chromatograms.<br>MestReNova version 9.0.1-13254 for compounds structure characterization.<br>Thermo Xcalibur version 3.0.63 for LC-ESI-HRMS data analysis.<br>ChemBioDraw version 14.0.0.117 for drawing compounds structure.<br>BioEdit version 7.2.6 for sequence alignment.

For manuscripts utilizing custom algorithms or software that are central to the research but not yet described in published literature, software must be made available to editors and reviewers. We strongly encourage code deposition in a community repository (e.g. GitHub). See the Nature Portfolio guidelines for submitting code & software for further information.

Data

Policy information about availability of data

All manuscripts must include a data availability statement. This statement should provide the following information, where applicable:

- Accession codes, unique identifiers, or web links for publicly available datasets
- A description of any restrictions on data availability
- For clinical datasets or third party data, please ensure that the statement adheres to our policy

The data generated in this study are provided in the Supplementary Information. The genome sequence of S. coelicolor M145 was sourced from NCBI (Reference Sequence ID: NC_003888.3). The NGS, WGS, and RNA-seq data for S. coelicolor M145 generated in this study have been deposited in the NCBI Sequence Read Archive database under accession code PRJNA1064385 [https://www.ncbi.nlm.nih.gov/sra/?term=PRJNA1064385]. The WGS data for S. avermitilis 3-115 and its derivative mutants generated in this study are available in the NCBI Sequence Read Archive database under accession code PRJNA1066248 [https://www.ncbi.nlm.nih.gov/sra/?term=PRJNA1066248]. Other data supporting the findings of this study are included in the published article and Supplementary Information. Requests for any additional information can be made to the corresponding authors. Source data are provided in this paper.

Research involving human participants, their data, or biological material

Policy information about studies with human participants or human data. See also policy information about sex, gender (identity/presentation), and sexual orientation and race, ethnicity and racism.

Reporting on sex and gender	Not applicable.
Reporting on race, ethnicity, or other socially relevant groupings	Not applicable.
Population characteristics	Not applicable.
Recruitment	Not applicable.
Ethics oversight	Not applicable.

Note that full information on the approval of the study protocol must also be provided in the manuscript.

Field-specific reporting

Please select the one below that is the best fit for your research. If you are not sure, read the appropriate sections before making your selection.

☒ Life sciences ☐ Behavioural & social sciences ☐ Ecological, evolutionary & environmental sciences

For a reference copy of the document with all sections, see nature.com/documents/nr-reporting-summary-flat.pdf

Life sciences study design

All studies must disclose on these points even when the disclosure is negative.

Sample size	About 100-500 exconjugants on the plate were randomly picked for comprehensively investigation of editing efficiency and off-target effects. The mixture genomes was extracted and sent for sanger sequencing or Illumina sequencing.
Data exclusions	No data was excluded.
Replication	Three biological and technical replicates were evaluated with positive for related experiments unless indicated.
Randomization	Exconjugants for sanger sequencing or Illumina sequencing were randomly picked.
Blinding	Blinding was not performed in this study, because the data collection or analysis are not susceptible to biased interpretation.

Reporting for specific materials, systems and methods

We require information from authors about some types of materials, experimental systems and methods used in many studies. Here, indicate whether each material, system or method listed is relevant to your study. If you are not sure if a list item applies to your research, read the appropriate section before selecting a response.

Materials & experimental systems

n/a	Involved in the study
☒	☐ Antibodies
☒	☐ Eukaryotic cell lines
☒	☐ Palaeontology and archaeology
☒	☐ Animals and other organisms
☒	☐ Clinical data
☒	☐ Dual use research of concern
☒	☐ Plants

Methods

n/a	Involved in the study
☒	☐ ChIP-seq
☒	☐ Flow cytometry
☒	☐ MRI-based neuroimaging

Plants

Seed stocks	Not applicable.
Novel plant genotypes	Not applicable.
Authentication	Not applicable.
